# Parallel DNA polymerase chain reaction: Synthesis of two different PCR products from a DNA template

**DOI:** 10.12688/f1000research.5813.1

**Published:** 2014-12-31

**Authors:** Vikash Bhardwaj, Kulbhushan Sharma

**Affiliations:** 1 Molecular Biology and Genetics Domain, Lovely Professional University, Punjab, 144411, India; 2 MCS Group, Institute of Nuclear Medicine and Allied Sciences (INMAS), Timarpur, Delhi, 110054, India

## Abstract

Conventionally, in a polymerase chain reaction (PCR), oligonucleotide primers bind to the template DNA in an antiparallel complementary way and the template DNA is amplified as it is. Here we describe an approach in which the first primer binds in a parallel complementary orientation to the single-stranded DNA, leading to synthesis in a parallel direction. Further reactions happened in a conventional way leading to the synthesis of PCR product having polarity opposite to the template used. This is the first study showing that synthesis of DNA can happen also in a parallel direction. We report that from a single-stranded DNA template, two different but related PCR products can be synthesized.

## Introduction

**Figure 1.**
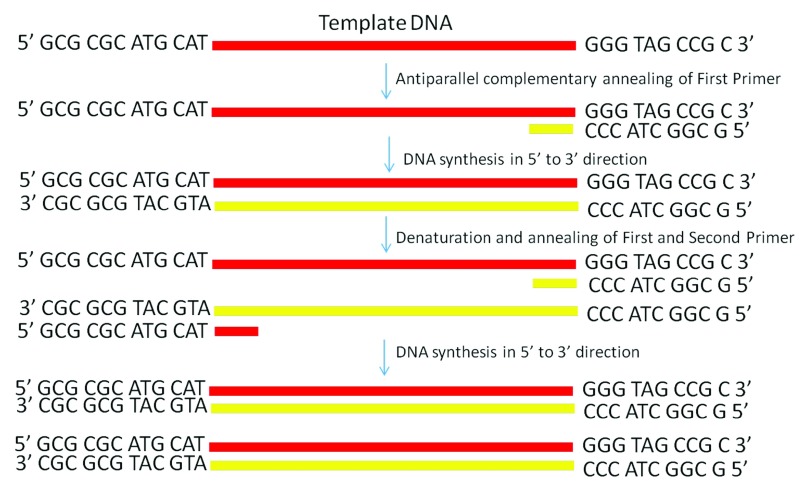
Schematic diagram showing PCR amplification of a single-stranded DNA by using conventional antiparallel oligonucleotide primers.

Our fundamental knowledge of DNA structure is based on the Watson-Crick model of DNA double helix, in which two polynucleotide chains running in opposite direction are held together by hydrogen bonds between the nitrogenous bases. Guanine can bind specifically only to cytosine (G-C) whereas adenine can bind specifically to thymine (A-T). These reactions are described as base pairing and the paired bases are said to be “complementary” ^1^. Conformational polymorphism of DNA is now extending beyond the Watson-Crick double helix. In 1986, using forced field calculation for a short ‘A-T’ rich DNA, Pattabiraman proposed the hypothesis that homopolymeric duplex DNA containing d(A)6d.(T)6 can form a thermodynamically stable parallel right-handed duplex DNA with reverse Watson-Crick base pairing. He also reported that the number and type of hydrogen bonds between A-T base pair are the same as that of antiparallel double helix ^2^. In 1988, the experimental strategies by Ramsing and Jovin confirmed that DNA containing A-T base pairs can exist as a stable parallel-stranded helix. The “Tm” value of both PS-DNA (parallel-stranded DNA) and APS-DNA (antiparallel-stranded DNA) showed a classical dependence upon salt concentration. They reported that at any given NaCl concentration, the melting temperature of PS-DNA was 15°C lower than its APS-DNA counterpart. In 2 mM MgCl _2_, the melting temperature for PS-DNA and APS-DNA was reported approximately same as those obtained in 0.2–0.3 M NaCl, demonstrating pronounced stabilization afforded by divalent cations ^3^. A similar study by Sande *et al.* on hairpin deoxyoligonucleotides having oligonucleotides sequence in parallel polarities (PS-hairpin) also confirmed the existence of parallel-stranded conformation. They have shown that parallel-stranded hairpins form stable duplex and get denatured at 10°C lower than corresponding APS oligomers ^4^. These two experimental studies provided evidence that DNA containing “A-T” base pairs can form both PS-DNA and APS-DNA. In 1992, Tchurikov *et al.* showed that parallel complementary probes of normal nucleotide consisting of both AT/GC base pairs can be used for molecular hybridization experiments, indicating the stability of G-C containing parallel DNA ^5^. In 1993, Borisova *et al.* reported that G-C pairs in a 40 base pair parallel duplex DNA (consisting of natural DNA sequence) are more thermostable than A-T base pairs ^6^. Furthermore, other similar reports have shown that there are no drastic differences in nearest neighbor base pair interactions between PS-DNA and APS-DNA having mixed AT/GC composition ^7^. The specificity of the interaction between the strands in parallel DNA has also been studied and it is so high that parallel probe as short as 40 nucleotide length is able to detect a specific band in Southern blot hybridizations on whole genome DNA ^8^. The polymerase chain reaction (PCR) developed by Mullis consists of denaturation of double-stranded DNA, primer annealing and extension. The process is repeated multiple times and the template DNA is amplified millions of times without any change in polarity of DNA ^9^ ( Figure 1). In 2000, Veitia and Ottolenghi reported that several attempts to amplify L15253 by PCR using different pairs of primers were unsuccessful. They suggested that there are no thermodynamic constraints which will prevent parallel nucleic acid synthesis, and the deoxynucleotide triphosphates used for a normal antiparallel polymerization reaction can also serve for a parallel reaction, provided that the polymerase enzyme is capable in catalyzing the nucleophilic interaction between the 3´OH and a 5´PPP from nucleotides arranged in a parallel way with respect to the template DNA ^10^.

**Figure 2.**
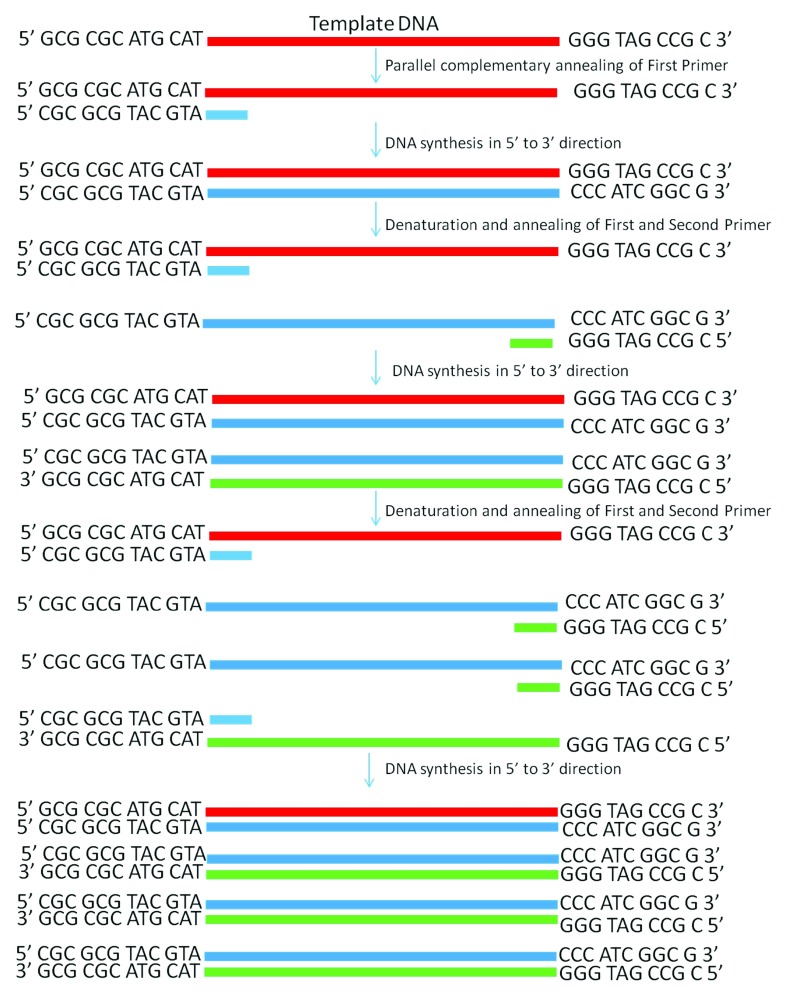
Schematic diagram showing PCR amplification of a single-stranded DNA by using the PD-PCR (parallel DNA PCR) approach in which the first primer binds to the template DNA in a parallel complementary manner. The second primer binds to the newly synthesized DNA in an antiparallel manner and later both primers amplify the new DNA in a conventional manner. PCR products obtained will have opposite polarity as compared to the template used.

In this study, we explored whether parallel DNA synthesis is feasible. We proposed the hypothesis that this reaction can be possible if we start a reaction using single stranded DNA as a template. We have shown that the Taq DNA polymerase can even extend the oligonucleotide primer annealed to single stranded DNA in a parallel complementary manner. The details of how our proposed parallel DNA PCR differs from the conventional PCR is shown in Figure 1 and Figure 2.

## Materials and methods

### PCR

PAGE purified single stranded DNA of 120 bp was commercially obtained at a scale of 1 O.D. from Sigma Aldrich, USA. PCR oligonucleotide primers were also purchased at a scale of 0.05 O.D. from Sigma Aldrich. The sequence of custom synthesized template DNA and oligonucleotide primers used in the study are shown in Table 1. In the PD-PCR reaction, we used (PD-PCR-1) and (PD-PCR-2) primer set while for conventional PCR we used (PCR-1) and (PCR-2) primers (see Table 1). Rest of the reaction remained same. The details of PCR reaction mix were as follows: total reaction mix=50µl, primers=1µl each (50 picomole), Taq DNA polymerase=0.5µl (5U/µl), dNTP mix=0.5µl (10mM), 10X PCR buffer=5µl, water=39µl and template DNA=3µl (0.114 ng). Taq DNA polymerase (M0273S) and dNTP mix (N0447S) were purchased from NEB (New England Biolabs). PCR analysis was performed using *Veriti* ^®^ Thermal Cycler (Applied Biosystem) by taking single stranded template DNA and amplifying it for 30 cycles at varying annealing temperature *viz.* 45°C, 50°C, 55°C, 58°C, 60°C, 65°C. PCR programming included 30 cycles of denaturation at 95°C for 15 seconds, annealing at varying temperatures for 30 seconds (as explained above) and extension at 72°C for 30 seconds.

**Table 1.** Shows sequence of custom synthesized template DNA and oligonucleotide primers used in this study.

Sequence of single stranded template DNA	5´ GCG CGC ATG CAT GTG ACT GAC GAT CGA TCG ATC AGT ACT GAC TGA CAA ATG ACT GGA TCC GGG AAG CTT GTG TTT AAA GTG TGA GGG TTG GCT GGG GTG TGG GG G TG GAT GGG TAG CCG C 3´
Oligonucleotides primers for conventional PCR ( Figure 1)	(PCR-1) 5´GCG GCT ACC CAT CCA CCC CCA C 3´ (PCR-2) 5´ GCG CGC ATG CAT GTG ACT GAC 3´
Oligonucleotides primers for PD-PCR ( Figure 2)	(PD-PCR-1) 5´CGC GCG TAC GTA CAC TGA CTG C 3´ (PD-PCR-2) 5´ CGC CGA TGG GTA GGT GG GGG 3´

### Agarose gel electrophoresis

PCR products obtained in two reactions were separated on 1% agarose gel containing ethidium bromide and were run and observed in gel-doc (DNR Bioimaging system, Jerusalem, Israel) under UV. Electrophoresis apparatus used in these experiments was purchased from Chromus Biotech, Bengaluru, India whereas chemicals {Agarose ( *A9539*) and EtBr ( *E7637*)} were purchased from Sigma, USA.

### Sequencing

The amplified products were sequenced at Eurofins Genomics India Pvt. Ltd. Karnataka India.

### Real time PCR

Real time PCR was performed with 2X SYBR Green master mix (K0221, Thermo Scientific, Pittsburgh, USA). The details of real time PCR reaction mix were as follows: total reaction mix=10µl, primers=0.25µl each, (50 picomole), 2X SYBR green mastermix=5µl, water=4µl and template=0.5µl (1:1000 dilution from original stock of 0.96 picomole). In the PD-PCR reaction, we used (PD-PCR-1) and (PD-PCR-2) primer set while for conventional PCR we used (PCR-1) and (PCR-2) primers (see Table 1). Negative control included reaction mix without template DNA. Reactions were incubated at 94°C for 5 minutes, followed by 30 PCR cycles of 94°C for 15 seconds, 50°C for 30 seconds and 72°C for 60 seconds using Mx3005P qPCR System - Agilent Technologies, Inc. The data were analyzed by using 2 ^−ΔCt^ method. The products were also run on agarose gel and visualised on gel doc system as previously described.

## Result and discussion

DNA sequencing file for PCR and PD-PCR DNA sequencing file (DNA sequencing file for PCR.ab1) confirming single stranded template DNA was amplified as it is while DNA sequencing file (DNA sequencing file for PD-PCR.ab1) confirming that single stranded template DNA was amplified as per our proposed PD-PCR scheme within this manuscript.

Real time PCR file for PCR and PD-PCR Real time PCR amplification of single stranded DNA as per conventional PCR and PD-PCR.1 Data file

**Figure 3.**
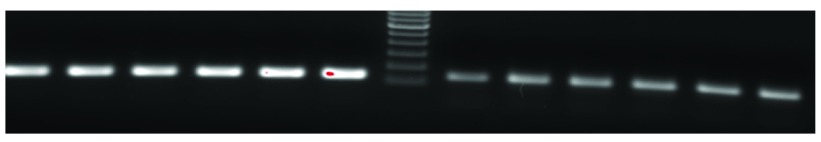
PD-PCR (parallel DNA PCR) and PCR: Lanes 1–6 show 120 bp PCR products amplified at annealing temperature of 45°C, 50°C, 55°C, 58°C, 60°C, 65°C, respectively, using conventional antiparallel complementary primers. Lane 7 is 100 bp molecular weight marker and Lanes 8–13 show PCR products amplified by parallel DNA PCR scheme at annealing temperature of 45°C, 50°C, 55°C, 58°C, 60°C, 65°C, respectively. In all cases, denaturation was performed at 95°C for 15 seconds, annealing for 30 seconds while extension at 72°C for 30 second for a total of 30 cycles.

**Figure 4.**
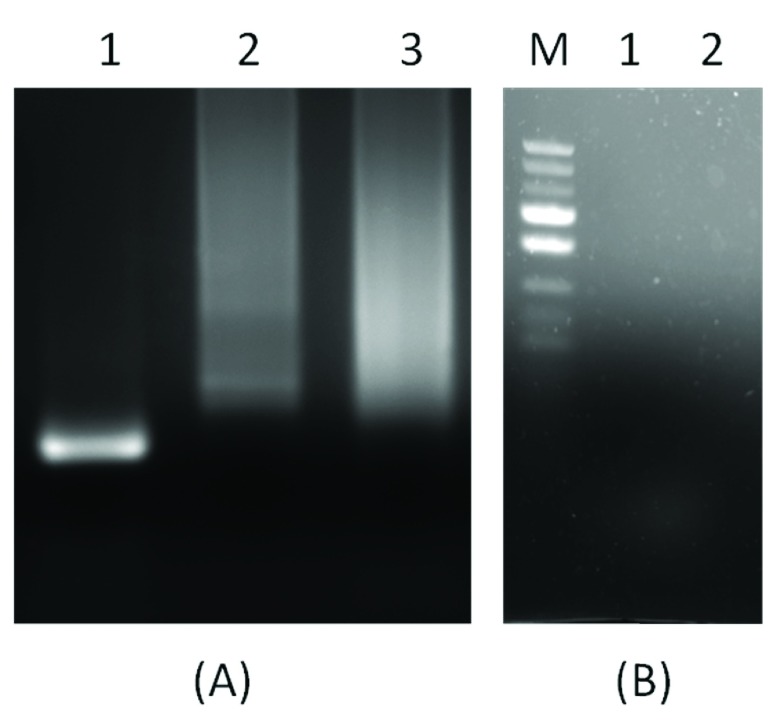
( **A**): A control reaction showing that PCR products were obtained when both primers were added as per scheme in Figure 2. In Figure 4 (A), Lane 1 shows 120 bp PCR products synthesized by PD-PCR, while in Lanes 2 and 3, only single primers were added and as expected no PCR product was synthesized. Figure 4(B) shows a negative control reaction of conventional PCR and PD-PCR in which the template DNA was not added.

**Figure 5.**
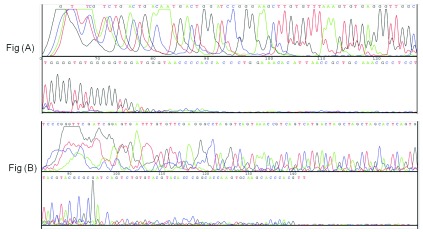
DNA sequencing results. Sequencing results in ( **A**) show that 120 bp DNA was amplified as it is while sequencing results in ( **B**) confirm that PCR products were obtained as per the scheme shown in Figure 2.

**Figure 6.**
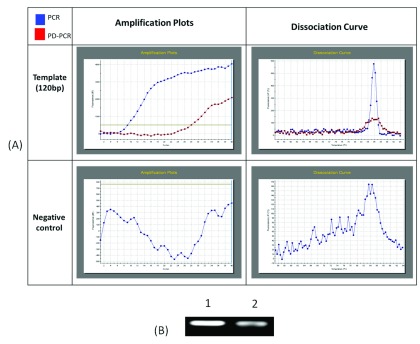
Real time PCR and PD-PCR ( **A**) show ampliflication plot and dissociation curves obtained after real time PCR analysis of amplification of 120 nucleotides single stranded template DNA via conventional PCR and PD-PCR. In control reaction no template DNA was added. ( **B**) PCR products obtained in real time PCR were also run on agarose gel and visualised on gel doc system.

The thermal denaturation analysis of parallel DNA has shown that it melts at a lower temperature than the corresponding antiparallel structure ^3, 4^. This finding gives us the clue that using double-stranded antiparallel DNA as a template for PD-PCR will not be possible as during annealing steps, antiparallel double-stranded DNA will anneal to itself without binding to parallel-stranded complementary primers. To avoid this, we started our PCR with a single-stranded DNA template. Details on how our proposed parallel DNA PCR (PD-PCR) differs from conventional PCR are shown in Figure 2. The first oligonucleotide primer (PD-PCR-1) was designed to bind the single-stranded template DNA in a parallel complementary manner. The parallel complementary annealing of the first primer allowed the synthesis of DNA in a parallel direction to the single-stranded DNA template. After the first denaturation step, the second oligonucleotide primer (PD-PCR-2) was designed to anneal to the newly synthesized DNA in an antiparallel complementary orientation. Further, both first and second primers used in this reaction amplified the new second DNA strand in a conventional way by binding in an antiparallel complementary way. Figure 3 (lanes 8–13) shows a 120 bp PCR product amplified by parallel DNA PCR scheme at annealing temperature of 45°C, 50°C, 55°C, 58°C, 60°C, 65°C respectively. In all cases, denaturation was performed at 95°C for 15 seconds, annealing for 30 seconds while extension at 72°C for 30 second for a total of 30 cycles. Similarly, as a control reaction, the single-stranded 120 bp DNA was amplified by conventional PCR in which the first primer (PCR-1) bound to the template DNA in an antiparallel orientation and the second primer (PCR-2) annealed to the newly synthesized DNA in an antiparallel orientation. Figure 3, Lanes 1–6 shows a 120 bp product PCR amplified at annealing temperature of 45°C, 50°C, 55°C, 58°C, 60°C, 65°C respectively using conventional antiparallel complementary primers. As a control reaction, PD-PCR was also performed using only one of the two primers. As expected, no PCR products were obtained ( Figure 4A lane 2 and 3). As a control reaction, conventional PCR and PD-PCR were performed without adding any template DNA. As expected, no PCR product was obtained confirming that no primer dimer was formed during both conventional PCR and PD-PCR ( Figure 4B). The DNA sequencing results confirmed that DNA templates were amplified in two different PCR products. Conventional PCR amplified the template DNA in its original orientation ( Figure 5A) whereas PD-PCR products read in a parallel direction to the template DNA ( Figure 5B). Primers and template used to show feasibility of PD-PCR till now ( Figure 3 and Table 1) were further used to perform real-time PCR. For this, a master mix containing SYBR Green and other components (except template and primers) was used. Primers and template were added to the master mix to make a final volume of 10µl. A Ct value of 9.26 was obtained for conventional PCR, 23.29 for PD-PCR whereas 33.15 was observed in negative control (without adding template DNA) indicating amplification in both conventional PCR and PD-PCR reactions ( Figure 6). The amplification indicated by Ct value in real time PCR was also confirmed by running the product on agarose gel ( Figure 6, lower panel). Weak amplifications in PD-PCR may be attributed to the fact that the actual amplification in conventional PCR is one step ahead than the PD-PCR. The first amplification in conventional PCR starts as early as denaturation followed by annealing ( Figure 1). On the other hand, in PD-PCR a new template is first synthesized during the first amplification (represented by green color in Figure 2). Once the template is ready, the conventional PCR goes on. Therefore, the amplified product shows low intensity as compared to the conventional PCR products. Taking together, our study has shown that DNA synthesis can happen in a parallel direction and two different, but related PCR products can be synthesized from the single-stranded template DNA. We hope that more molecular biology techniques will develop in future based on parallel complementary bindings of duplex DNA.

## Data availability

*F1000Research:* Dataset 1. DNA sequencing file for PCR and PD-PCR. 10.5256/f1000research.5813.d41515 ^11^

*F1000Research:* Dataset 2. Real time PCR file for PCR and PD-PCR. 10.5256/f1000research.5813.d41516 ^12^
