# Supplementary material for: Parallel DNA polymerase chain reaction: Synthesis of two different PCR products from a DNA template
Source: F1000Res. 2014 Dec 31;3:320. [Version 1] doi: 10.12688/f1000research.5813.1 (PMC4304224; doi:10.12688/f1000research.5813.1)
Supplement: Peer review history

# Peer review: Referee response for version 1

**DOI:** 10.5256/f1000research.6214.r7174

**Published:** 2015-01-19

**Authors:** Ram Gopal

**Affiliations:** 1 Department of Cell and Molecular Biology, Uppsala University, Uppsala, Sweden

It is interesting to read the article ‘Parallel DNA polymerse chain reaction: Synthesis of two different PCR products from a DNA template’ by Bhardwaj and Sharma. The authors for the first time have demonstrated that primers can anneal in parallel orientation and can be extended in a PCR reaction. They have used state of the art technique real time PCR to demonstrate this along with the conventional agarose gel electrophoresis and DNA sequencing reaction. Definitely, this opens up many possibilities in the field of molecular biology. The research is well designed and presented in a clear and comprehensible way. The introduction section gives a very nice insight. However, I suggest following minor changes to improve the manuscript.

 

1. Figure 3- Numbering should be done on the loading wells.
2. Figure 5- Sequencing results should be presented in such a way that one can easily compare the APS and PS sequences easily. For example they can put a arrow mark from where to compare.
3. Figure 6- It is difficult to read the abscissa and ordinate scale and naming. Increasing the font will help.
4. Line 11- d(A)6d.(T)6 should be explained or  is it dA _6 _dT _6  _?
5. In the RT PCR section the term Ct value should be explained so that a non-expert reader can benefit.
6. There are some typos errors scattered in the manuscript. Such as 50µl should be 50 µL.

I have read this submission. I believe that I have an appropriate level of expertise to confirm that it is of an acceptable scientific standard.

# Peer review: Referee response for version 1

**DOI:** 10.5256/f1000research.6214.r7172

**Published:** 2015-01-12

**Authors:** Harishkumar Madhyastha

**Affiliations:** 1 Department of Applied Physiology, Faculty of Medicine, Miyazaki Medical College, Miyazaki, Japan

The title of the research paper is well justified and suitable to the content of the subject. Abstract of the paper is well written and concluded. Materials and method, data analysis and design of the experiment are commendable. In my opinion, the paper is well written and opens a new paradigm for researchers and finally, the conclusion arrived at shows the high standard of the scientific achievement. In all respects, the paper is highly novel and can be indexed.

I have read this submission. I believe that I have an appropriate level of expertise to confirm that it is of an acceptable scientific standard.
